# Highly aligned stromal collagen is a negative prognostic factor following pancreatic ductal adenocarcinoma resection

**DOI:** 10.18632/oncotarget.12772

**Published:** 2016-10-20

**Authors:** Cole R. Drifka, Agnes G. Loeffler, Kara Mathewson, Adib Keikhosravi, Jens C. Eickhoff, Yuming Liu, Sharon M. Weber, W. John Kao, Kevin W. Eliceiri

**Affiliations:** ^1^ Department of Biomedical Engineering, University of Wisconsin, Madison, WI, USA; ^2^ Laboratory for Optical and Computational Instrumentation, University of Wisconsin, Madison, WI, USA; ^3^ Morgridge Institute for Research, Madison, WI, USA; ^4^ Department of Surgical Pathology, University of Wisconsin, Madison, WI, USA; ^5^ Department of Biostatistics and Medical Informatics, University of Wisconsin, Madison, WI, USA; ^6^ Department of Surgery, University of Wisconsin, Madison, WI, USA; ^7^ University of Wisconsin Carbone Cancer Center, Madison, WI, USA

**Keywords:** collagen, stroma, microenvironment, quantitative pathology, pancreatic cancer

## Abstract

Risk factors for pancreatic ductal adenocarcinoma (PDAC) progression after surgery are unclear, and additional prognostic factors are needed to inform treatment regimens and therapeutic targets. PDAC is characterized by advanced sclerosis of the extracellular matrix, and interactions between cancer cells, fibrillar collagen, and other stromal components play an integral role in progression. Changes in stromal collagen alignment have been shown to modulate cancer cell behavior and have important clinical value in other cancer types, but little is known about its role in PDAC and prognostic value. We hypothesized that the alignment of collagen is associated with PDAC patient survival. To address this, pathology-confirmed tissues from 114 PDAC patients that underwent curative-intent surgery were retrospectively imaged with Second Harmonic Generation (SHG) microscopy, quantified with fiber segmentation algorithms, and correlated to patient survival. The same tissue regions were analyzed for epithelial-to-mesenchymal (EMT), α-SMA, and syndecan-1 using complimentary immunohistostaining and visualization techniques. Significant inter-tumoral variation in collagen alignment was found, and notably high collagen alignment was observed in 12% of the patient cohort. Stratification of patients according to collagen alignment revealed that high alignment is an independent negative factor following PDAC resection (*p* = 0.0153, multivariate). We also found that epithelial expression of EMT and the stromal expression of α-SMA and syndecan-1 were positively correlated with collagen alignment. In summary, stromal collagen alignment may provide additional, clinically-relevant information about PDAC tumors and underscores the importance of stroma-cancer interactions.

## INTRODUCTION

Pancreatic ductal adenocarcinoma (PDAC) consistently ranks as one of the deadliest human cancers and projects to become the second leading cancer killer by 2030 [1]. Of all cancers, it continues to have the lowest 5-year survival rate of <6% due to a combination of factors, including the lack of an early screening or detection technique, vague symptomology, advanced stage at diagnosis, high recurrence, and suboptimal response to standard treatments [2]. In current PDAC management, radical surgical resection paired with adjuvant chemotherapy is the only potential curative treatment strategy. Although many PDAC cases are diagnosed at a locally-advanced or metastatic stage, 20% of patients with confined disease are eligible for surgery. For those that undergo resection, long-term survival (>5 years) is possible but reported in less than 25% of patients due to rapid local relapse or the emergence of micrometastases undetectable at the time of surgery [3–7]. Currently, risk factors for disease progression after surgery remain unclear. A positive surgical margin has been demonstrated to be a poor prognostic factor and is commonly utilized to guide current clinical surveillance and decisions; however, this and other classic prognostic factors such as tumor grade and stage do not consistently account for differences in patient survival [4,8].

One of the great ongoing clinical needs in PDAC management is to identify new tissue-based biomarkers that can predict aggressive disease behavior and patient prognosis. Until recently, both basic researchers and clinicians have focused primarily on the morphological and genetic features of PDAC cells for biological insight and the identification of potential clinical biomarkers [6,9–13]. One of the most striking characteristic features of PDAC is the presence of a dense desmoplastic stroma, which now is greatly appreciated and being studied in concert with the malignant epithelial component of PDAC. On average, the epithelial component of PDAC constitutes only 20-30% of the tumor volume, with the remainder composed of stromal cells, extracellular matrix (ECM) components, and soluble signaling mediators [14,15]. Stromal cells include fibroblasts, myofibroblastic pancreatic stellate cells (PSCs), endothelial cells, immune cells, adipose cells, and nerve cells. Key ECM components include fibrillar collagens [16–19], non-fibrillar collagens [20,21], fibronectin [22], periostin [23], tenascin-C [24,25], and others, all of which are aberrantly produced by cancer-associated fibroblasts (CAFs) and PSCs. As in many other solid tumor types, stromal biology has been shown to play a major role in PDAC progression by reciprocally interacting with cancer cells at all stages of the metastatic cascade, as well as by influencing therapeutic resistance [26–31].

Given that the PDAC stroma is a disease hallmark, several investigations have sought to determine if biomarkers harbored by the cancer-associated microenvironment may associate with tumor aggressiveness and patient prognosis [15,20,32–40]. A significant number of compelling reports have demonstrated that the specific organization of the stromal ECM, notably that of the fibrillar collagen landscape, plays a determinant role in tumor progression [41–43]. Interstitial fibrillar collagens have a characteristic triple helical structure, composed of three polypeptide alpha chains, which confers high tensile strength and an ideal structural support scaffold for tissue integrity. By exploiting the ability to detect and visualize fibrillar collagens with a number of techniques including Second Harmonic Generation (SHG) imaging [44–49], it is now appreciated that the normal architectural integrity of fibrillar collagen networks is lost during cancer progression. This has been shown to result in a number of biological consequences, such as tissue stiffening and the transmission of mechanical signaling [50–52], altered metabolic profiles [53], immune cell exclusion from the primary tumor [54], and permissive landscapes for cell invasion [41,55]. Furthermore, the detection of specific collagen topology changes in histopathological examination of tissue sections has demonstrated clinical value in augmenting diagnostics and predicting patient survival [42,43,56].

Fibrillar collagen is strongly implicated in PDAC progression due its remarkable abundance throughout the stroma [57,58]. We have recently shown that the PDAC-associated stroma is not only rich in fibrillar collagen amount, but also that collagen is reorganized and more aligned compared to that in benign pancreatic stroma [59]. The purpose of this study was to determine if the level of stromal collagen alignment can provide a quantifiable PDAC disease marker that correlates with patient prognosis following surgical resection. To do this, we specifically imaged fibrillar collagen in the stroma adjacent to the malignant epithelium in clinical histopathology specimens with SHG microscopy and quantified alignment using established fiber tracking algorithms. Furthermore, we mapped and evaluated the expression of the epithelial-to-mesenchymal (EMT) phenotype and established stromal cell markers (α-SMA, syndecan-1) to the same malignant regions to shed light on potential relationships between important components of the PDAC microenvironment.

## RESULTS

### SHG imaging reveals collagen alignment is elevated around PDAC cells and predicts poor survival

**Figure 1:**
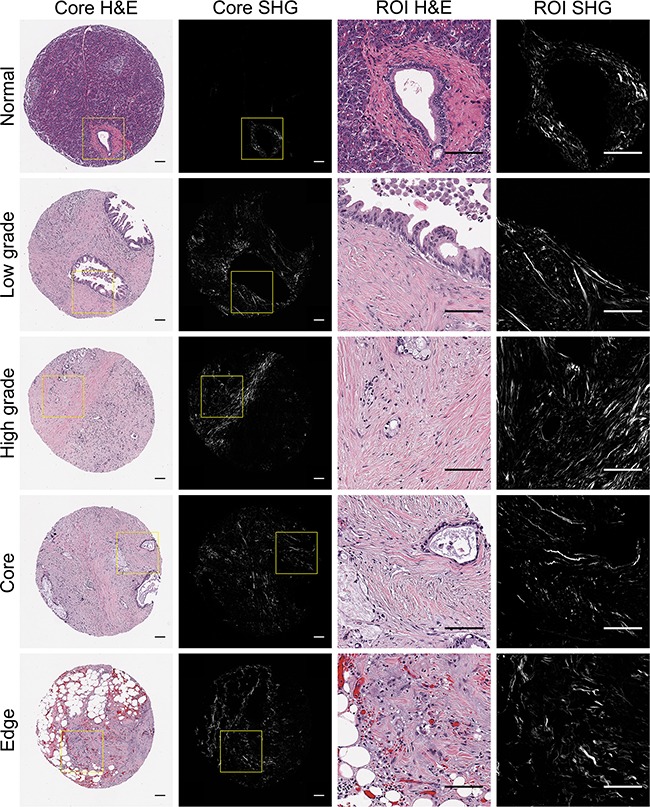
Representative pancreatic tissues visualized with H&E staining and SHG microscopy H&E stained tissues cores representing normal and distinct PDAC regions (low grade, high grade, tumor core, infilitrating edge) were visualized holistically with SHG stitching. For each core, 1-2 pathology-confirmed regions of interest (ROIs) containing malignant cells and associated stroma were marked to omit stroma associated with non-malignant tissue components. Yellow boxes indicate representative ROI regions that were extracted from whole TMA cores and subsequently quantified for collagen alignment.

SHG imaging is a nonlinear optical technique that can specifically image non-centrosymmetric harmonophores, such as fibrillar collagen [48], and has emerged as an indispensable tool to interrogate changes in both live, dynamic models [60–64] and clinical histopathologic slides [43,56,59]. Since the TMA used in this study contained multiple cores per patient, we were able to image and quantify collagen alignment in normal and distinct PDAC regions (low grade, high grade, tumor core, and infiltrating edge). As expected, stromal collagen was more aligned around PDAC cells compared to the random, less aligned organization around normal ducts (*p* < 0.05, Figure 1, Supplementary Figure S1A). There was no significant difference between the degree of collagen alignment around PDAC cells of low and high histological grade (*p* = 0.301, Supplementary Figure S1B), or in the tumor core versus the infiltrating edge (*p* = 0.061, Supplementary Figure S1C).

**Figure 2:**
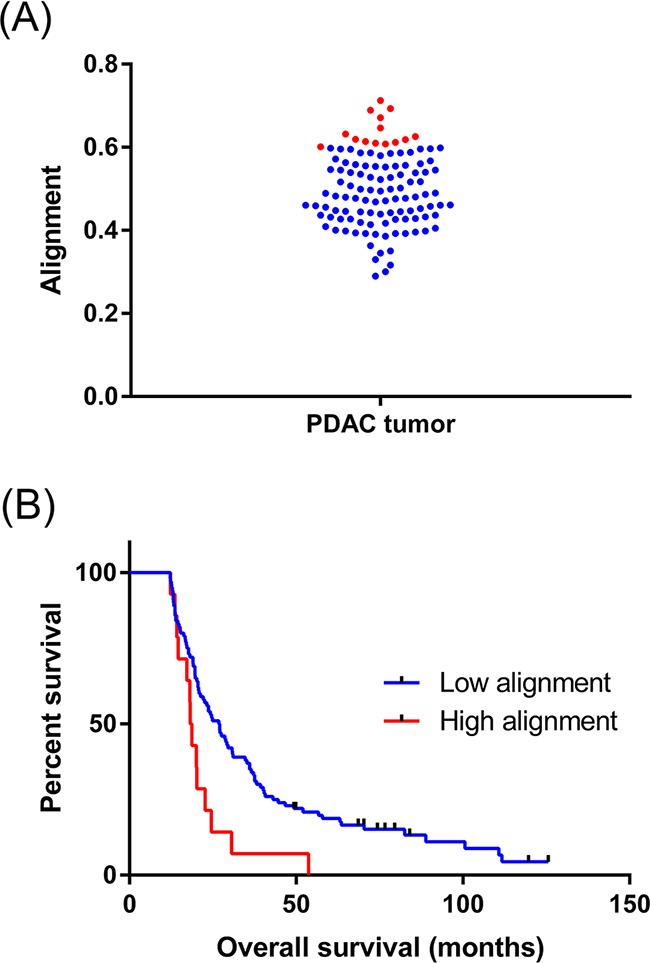
Collagen alignment significantly varies between individual PDAC tumors **A.** Different collagen alignment values for the entire 114 patient cohort. Data points highlighted in red represent highly aligned tumors. **B.** Kaplan-Meier analysis shows the post-surgical survival of patients with high collagen alignment is decreased. The median survival of each patient cohort was compared using the Log-rank test (*p* = 0.0062, univariate).

Although collagen alignment was elevated in PDAC tissues compared to normal, significant variation was observed among individual patient tumors (range 0.289 – 0.712, Figure 2A). Using the well-referenced X-tile software approach on a separate training patient cohort [65], we determined an optimal alignment cut-off to be ≥0.60. This was subsequently validated to be significant in our 114-patient experimental cohort, where 12% of the patients presented highly aligned collagen. No significant correlations were determined between common patient clinicopathological characteristics and collagen alignment with the exception of pT (*p* = 0.025, Table 1). PDAC patients with high collagen alignment had significantly reduced overall survival than the low alignment patients (median survival high alignment = 18.5 months, median survival low alignment = 26.9 months, HR: 2.25, *p* = 0.0062, univariate analysis, Figure 2B, Table 2). In multivariate analysis, it is noteworthy that many traditional predictors (*i.e.* margin status, stage, grade) were not significant in this patient cohort and may be a result of the selective patient inclusion criteria we imposed (*i.e.* no neoadjuvant therapy, inclusion of >5 year survivors). Also, gender was determined to be a significant prognostic factor, which was previously reported in a study based on a similar patient population [66]. Ultimately, high collagen alignment remained a negative prognostic factor of overall survival independent of traditional prognostic determinants (*p* = 0.0153, Table 3).

**Table 1:** Clinicopathological characteristics PDAC patients with low and high collagen alignment

Characteristic	Category	Low alignment (*n* = 100)	High alignment (*n* = 14)	*p*
Age (yr)	≤65	37	3	0.372
	>65	63	11	
Gender	Female	45	8	0.410
	Male	55	6	
Tumor location	Head	90	13	1.000
	Other	10	1	
Tumor size (cm)	≤2	23	1	0.295
	>2	76	13	
	Unknown	1	0	
pT	T1/T2	25	8	0.025
	T3/T4	74	6	
	Unknown	1	0	
pN	N0	26	7	0.111
	N1	74	7	
Stage	IA-IIA	25	7	0.065
	IIB-III	74	7	
	Unknown	1	0	
Grade	G1	20	3	1.000
	G2/G3	73	10	
	Unknown	7	1	
Venous invasion	No	52	7	0.437
	Yes	21	1	
	Unknown	27	6	
Lymphatic invasion	No	47	5	1.000
	Yes	26	3	
	Unknown	27	6	
Perineural invasion	No	20	2	1.000
	Yes	61	6	
	Unknown	19	6	

**Table 2:** Patient clinicopathological characteristics and univariate analysis of survival prediction

Characteristic	Category	*n*	median OS, mo	Comparison	HR	95% CI	*p*
Age (yr)	≤65	40	30.7	>65 *vs* ≤65	1.44	0.95 - 2.18	0.0839
	>65	74	21.0				
Gender	Female	53	20.0	Female *vs* Male	1.76	1.19 - 2.62	0.0051
	Male	61	30.8				
Tumor location	Head	103	24.5	Head *vs* Other	0.71	0.37 - 1.37	0.3036
	Other	11	20.9				
Tumor size (cm)	≤2	24	23.5	>2 *vs* ≤2	1.00	0.62 - 1.62	0.9951
	>2	89	24.5				
pT	T1/T2	33	24.5	T1/T2 *vs* T3/T4	1.08	0.70 - 1.65	0.7278
	T3/T4	80	24.0				
pN	N0	33	28.9	N0 *vs* N1	1.06	0.70 - 1.62	0.7724
	N1	81	22.7				
Stage	IA-IIA	32	26.6	IA-IIA *vs* IIB-III	1.08	0.68 - 1.73	0.7434
	IIB-III	81	23.7				
Grade	G1	23	30.6	G1 *vs* G2/G3	0.76	0.46 - 1.24	0.2672
	G2/G3	83	23.2				
Margin	R0	86	24.1	R0 *vs* R1	1.03	0.66 - 1.63	0.8843
	R1	28	23.4				
Venous invasion	No	59	24.9	No *vs* Yes	1.06	0.62 - 1.81	0.8428
	Yes	22	25.4				
Lymphatic invasion	No	52	28.0	No *vs* Yes	0.84	0.51 - 1.38	0.4874
	Yes	29	20.5				
Perineural invasion	No	22	29.2	No *vs* Yes	1.07	0.64 - 1.79	0.7913
	Yes	67	23.7				
Adjuvant therapy	No	45	23.7	No *vs* Yes	1.37	0.93 - 2.03	0.1159
	Yes	69	24.5				
Collagen alignment	Low	100	26.9	High *vs* Low	2.25	1.26 - 4.01	0.0062
	High	14	18.5				

**Table 3:** Final multivariate analysis of survival prediction

Characteristic	Comparison	HR	95% CI	*p*
Collagen alignment	High *vs* Low	2.20	1.63 - 4.14	0.0153
Gender	Female *vs* Male	1.91	1.24 - 2.96	0.0036
Adjuvant therapy	No *vs* Yes	1.45	0.94 - 2.53	0.0941
Age (yr)	>65 *vs* ≤65	1.25	0.80 - 1.94	0.3310
Margin	R1 *vs* R0	1.02	0.60 - 1.72	0.9561
Grade	G2/G3 *vs* G1	1.41	0.83 - 2.39	0.2085
pT	T3/T4 *vs* T1/T2	1.22	0.75 - 2.00	0.4224
pN	N1 *vs* N0	1.08	0.67 - 1.74	0.7578

### EMT expression by PDAC cells in the context of aligned collagen

**Figure 3:**
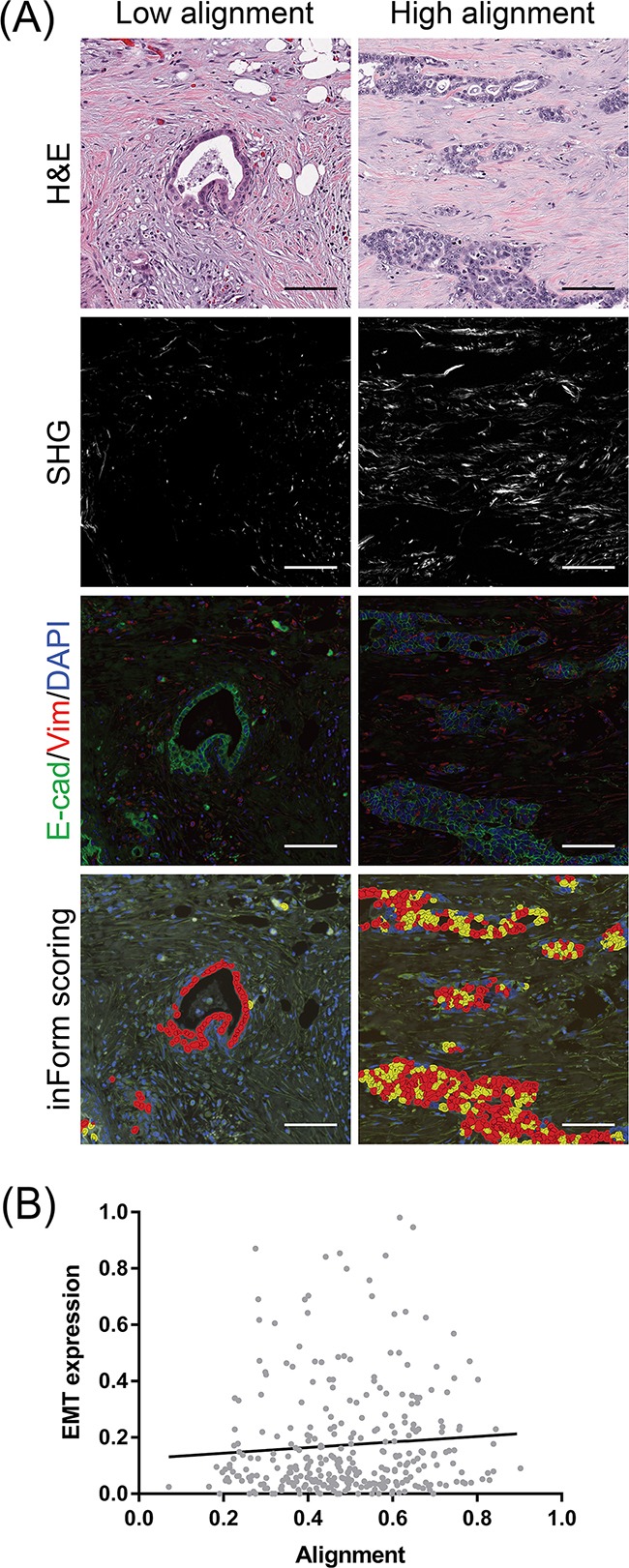
Co-localization of EMT-positive PDAC cells with stromal collagen alignment **A.** Sections adjacent to the H&E tissues from which SHG was measured were immunofluorescently labeled for E-cadherin, vimentin, and DAPI. The same regions were scored for double positivity using inForm analysis software as an indication of EMT (yellow overlays). A greater proportion of cells express EMT in the context of highly aligned collagen. Scale bars = 100 μm **B.** Correlation of EMT-expressing PDAC cells with the degree of collagen alignment. EMT expression indicates the fraction of total PDAC cells double positive for E-cadherin and vimentin. A total of 315 confirmed PDAC tissues cores representative of 114 patients were analyzed. Spearman r = 0.116, *p = 0.039

Experimentally, collagen organization appears to play a functionally important role in cancer cell migration and navigation through the stroma [41,62]. We therefore examined our PDAC patient cohort for the expression of the epithelial–mesenchymal transition (EMT) phenotype. We performed multispectral immunohistofluorescent imaging for E-cadherin and vimentin on adjacent tissue sections and scored double positivity in PDAC cells. EMT was not significantly different between low and high histological grade PDACs (*p* = 0.188, Supplementary Figure S2A). Spatially, a greater fraction of PDAC cells demonstrated EMT in the infiltrating edge of the tumor (0.210) versus the tumor core (0.172) (*p* = 0.043, Supplementary Figure S2B). Qualitatively, we noticed that infiltrating PDAC cell clusters co-expressing E-cadherin and vimentin appeared to localize to aligned regions of collagen (Figure 3A). By quantifying all confirmed PDAC tissue cores for E-cadherin/vimentin double positivity, we determined that there was a significant positive correlation between collagen alignment and EMT expression by PDAC cells (Spearman r = 0.202, *p* = 0.031, Figure 3B). There was no correlation between the total number of PDAC cells and collagen alignment (Spearman r = 0.023, *p* = 0.6893).

### Collagen alignment and CAF markers in the stroma neighboring PDAC cells

**Figure 4:**
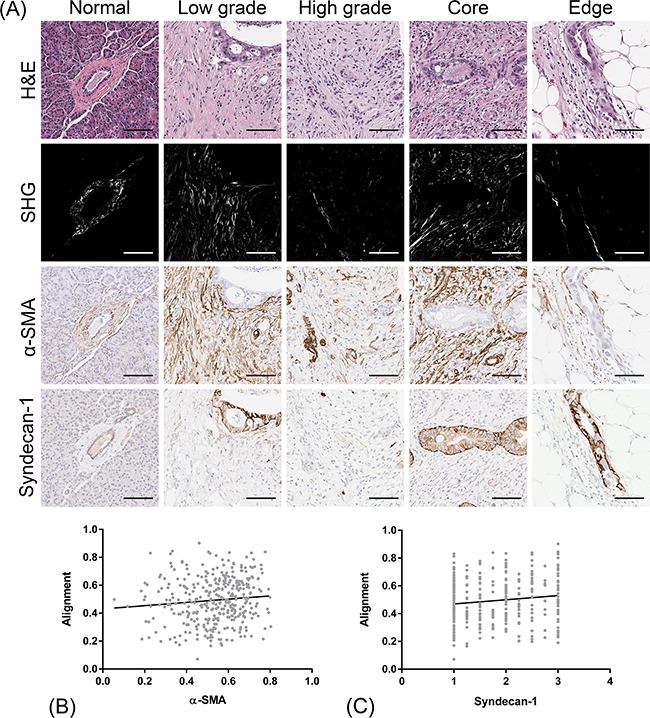
Co-localization of CAF markers (α-SMA and syndecan-1) with stromal collagen alignment **A.** Sections adjacent to the H&E tissues from which SHG was measured were immunohistochemically labeled for α-SMA and syndecan-1 and scored for stromal positivity. Both markers co-localize and orient along aligned collagen fibers. Scale bars = 100 μm **B.** Correlation of α-SMA expression with the degree of stromal collagen alignment in the periductal stroma of PDAC cells. Spearman r = 0.121, *p = 0.022. **C.** Correlation of syndecan-1 expression with the degree of stromal collagen alignment in the periductal stroma of PDAC cells. Spearman r = 0.115, *p = 0.029. A total of 356 confirmed PDAC tissues cores representative of 114 patients were analyzed.

Increasing evidence suggests a functional role of CAFs in PDAC aggressiveness [67]. To assess the relationship of collagen organization to these stromal cells, we visualized and quantified immunohistochemical positivity for α-SMA and syndecan-1 in the same regions from which collagen alignment was measured (Figure 4A). Most contractile myofibroblasts, notably pancreatic stellate cells (PSCs) in the context of PDAC, express α-SMA; consequently α-SMA is used as a surrogate marker for PDAC stromal activation in the experimental setting [33,30,40]. Since α-SMA is predominantly expressed in the stroma, we were able to assess positivity by color deconvolution in the same pathology-confirmed ROIs from which SHG measurements were acquired. Overall, minimal α-SMA positivity was detected in the normal stroma, whereas PDAC-associated stroma showed significantly higher expression (*p* < 0.0001, Supplementary Figure S3A). Periductal α-SMA expression was significantly higher in low grade tumors (0.599) compared to high grade tumors (0.499) (*p* = 0.008, Supplementary Figure S3B) and in the tumor core (0.572) compared to the tumor edge (0.503) (*p* = 0.0004, Supplementary Figure S3C). In the cancer-associated stroma, α-SMA-positive stromal cells appeared to orient along aligned collagen fibers (Figure 4A). To complement these qualitative observations, we determined that collagen alignment was positively correlated to α-SMA expression (Spearman r = 0.121, *p* = 0.022, Figure 4B).

Syndecan-1 is a transmembrane heparin sulphate proteoglycan that binds soluble factors and ECM components, and regulates cell growth, cell-cell and cell-matrix adhesion, and cell migration [68]. Syndecan-1 expression by CAFs has been reported in the progression of a number of cancers and has been implicated in ECM alignment [69]. Since syndecan-1 can be variably expressed by both epithelial and stromal compartments [70,71], we independently evaluated the stroma of the PDAC tumors on a nominal scale. In normal ductal epithelium, characteristic basolateral positivity was detected (Figure 4A). The cancer-associated stroma showed significantly elevated syndecan-1 levels compared to normal stroma (*p* < 0.0001, Supplementary Figure S4A). No significant differences were observed in stromal syndecan-1 expression between histological grade (Supplementary Figure S4B) or tumor region (Supplementary Figure S4C). Interestingly, though, collagen alignment was also positively correlated to stromal syndecan-1 expression (Spearman r = 0.115, *p* = 0.029, Figure 4C).

## DISCUSSION

There is increasing interest by PDAC researchers and clinicians alike to quantify stromal characteristics to gain a more integrated understanding of tumors. Consistent with other studies investigating the role of collagen in malignant tissues, we recently demonstrated that collagen alignment is elevated in the PDAC-associated stroma, distinguishing it from that of normal and chronic pancreatitis stroma [59]. To further investigate the potential clinical relevance of stromal collagen alignment in PDAC, we utilized SHG microscopy to image human PDAC tumor samples backed by extensive patient data. Using this approach, we first determined that collagen alignment is elevated in the cancer-associated stroma, which is in concordance with our previous results. While alignment did not differ significantly intra-tumorally, substantial heterogeneity was observed among patients and stratification revealed high collagen alignment as an unfavorable, independent prognostic factor in PDAC. This is an especially important insight given that PDAC generally has relatively short post-operative survival and few, weak clinical prognosticators after definitive surgery.

Our approach and findings support the potential utility of assessing PDAC collagen alignment in the context of clinical pathology. In the pre-operative setting, core biopsy material could be examined. A pre-operative biopsy may not always yield a large amount of collagen stroma, but if it did and high stromal collagen alignment predictive of aggressiveness was detected, that information could help the clinical team in deciding whether to operate within a particular clinical context, including lymph node involvement, a compromised celiac axis, comorbidities, or other perioperative factors that may impact surgical risk. Alternatively, collagen alignment could be characterized in resected tissue to assist clinicians in stratifying patients into prognostic subgroups and tailoring adjuvant management regimens.

To date, most collagen organization studies have been largely descriptive and have failed to investigate potential relationships of collagen alignment to the distribution and phenotypes of cells within the microenvironment. To complement our SHG findings, we correlated collagen alignment to protein expression data generated by advanced digital pathology technologies. EMT is a migratory program that may be triggered early in PDAC progression [7,72], and evidence points to EMT as an independent negative prognostic factor in PDAC [72,73]. Mechanistically, PDAC cells invade in response to collagen I by inducing EMT-related transcription factors [74–78]. Interestingly, our data showed that EMT expression by PDAC cells and collagen alignment were positively correlated. Aligned collagen and the EMT phenotype may be related features of aggressive tumors, reflecting a possibility where PDAC cells are utilize the stromal framework for efficient invasion.

Interestingly, tumor cell number and collagen alignment were not significantly correlated in our study suggesting a role of extrinsic influences. CAFs in the stroma modulate the progression of many cancers [79,80]. In PDAC, PSCs are the major CAF driving desmoplasia. Once activated, PSCs release a number of soluble mediators and have been identified as the primary source of fibrillar collagen deposition in the stroma [81,82]. To investigate the distribution of CAFs and potential association with aligned collagen, we systematically quantified stromal α-SMA and syndecan-1 expression in the same regions from which SHG and EMT measurements were acquired. α-SMA is a contractile protein up-regulated in myofibroblasts and is an established marker for PSC activation [83]. Additionally, α-SMA expression negatively correlates with PDAC survival [33, 39, 40]. Syndecan-1 is a transmembrane receptor that binds numerous ECM ligands, including collagen [46, 84]. The shift of syndecan-1 expression from the epithelium to the stroma is a poor prognostic factor in breast cancer [70] and PDAC [71]. Recent work has shown that stromal syndecan-1 expression induces ECM alignment in breast cancer, although the underlying mechanism remains unclear [69]. Our data shows that stromal α-SMA and syndecan-1 expression both show a significant positive correlation with collagen alignment, indicating a potential role of CAFs in ECM remodeling.

Our presented findings establish a strong foundation to further investigate the clinical value of collagen alignment in PDAC. While our study represents patient samples collected over many years at our institution, it will be of great interest to expand this work to patients from additional institutions and geographical locations. This would help clarify interesting observations of our particular study cohort, including gender as a significant prognostic factor and collagen alignment inversely correlating with the size and extent of the primary tumor (pT). Additionally, a prospective study designed to analyze larger tissue sections, in conjunction with molecular characterization of the primary tumor and known serological biomarkers, may yield additional information about clinically-relevant collagen changes. Although our data shows significant correlations between collagen alignment, α-SMA, syndecan-1, and EMT, this should be further confirmed mechanistically using human-derived tissues. It is also likely that other microenvironment factors (*i.e.* other cell types, ECM components, signaling molecules) play a role in collagen alignment that were not considered in our study, but remain active areas of research [18,19,25,32,43,50,62,85–95]. Taken together, quantification and further study of collagen alignment has the potential to provide additional, clinically-relevant information about PDAC tumors.

## CONCLUSION

Improved characterization of PDAC tumors is needed to predict prognosis, guide treatment, and enhance pathobiologic understanding of this deadly disease. Using a SHG-based quantitative approach, we show that high stromal collagen alignment is related to poor patient prognosis. Stromal collagen alignment also correlates with EMT and the distribution of activated CAFs. These findings provide initial insights on the role of collagen alignment in PDAC and its relationship to epithelial and stromal cells. The collective literature on the role of collagen in cancer progression and its repeatedly demonstrated correlation with patient outcomes in a variety of cancers underlines the need to develop robust prospective clinical studies to assess its value as a biomarker in the clinical setting. In addition, the relationship between collagen alignment, EMT and CAF appears to be critical to cancer progression but is not yet fully understood. Further studies are required to gain a greater integrated understanding of the PDAC microenvironment.

## MATERIALS AND METHODS

### Tissue microarray construction

A human PDAC tissue microarray (TMA) resource available at the University of Wisconsin Carbone Cancer Center was retrospectively studied. The TMA was constructed from patient tissues that underwent surgery with curative intent at the University of Wisconsin Hospital and Clinics between 1987 and 2012. For our particular investigation, it was crucial that none of the patients underwent neoadjuvant chemotherapy and/or radiotherapy, as evidence suggests that this can affect stromal activation and fibrosis [96–98]. All source tissue for the TMA was from archival, formalin fixed paraffin embedded (FFPE) blocks that were created for the purpose of routine diagnostic examination. Hematoxylin and eosin (H&E) sections prepared from these blocks were reviewed by board-certified pathologists to identify different representative tissue regions, including normal adjacent pancreas, the tumor core, and infiltrating tumor edge. Also, since it is relatively common for PDAC to have a combination of well and poorly-differentiated ducts in the same tumor, low and high histological grade regions were identified. Each different region, if represented, was marked on the H&E slides and the corresponding region in the block was punched using a 1 mm diameter core and inserted into a recipient FFPE block using a Beecher Instruments MTA-1 manual tissue arrayer. Therefore, each patient could be represented by up to 5 cores depending on tissue availability and quality.

### Histological staining and imaging

The TMA was serially sectioned at 5 μm thickness, mounted on charged slides, and deparaffinized. The first section was stained using standard H&E. The second section was co-stained for E-cadherin and vimentin immunohistofluorescence. Antigen retrieval was performed in citrate buffer at pH 6.0. Slides were blocked for 1 hour using 10% goat serum, then incubated with anti-E-cadherin (1:200; mouse monoclonal, clone 24E10, Cell Signaling) and anti-vimentin (1:6400, rabbit monoclonal, clone V9, Dako) overnight at 4°C. After washing, slides were incubated with goat anti-mouse Alexa Fluor 555 and goat anti-rabbit Alexa Fluor 488 (both 1:500) for 30 minutes, mounted with DAPI, and coverslipped. The third and fourth serial sections were stained for α-SMA (clone 1A4, CellMarque) and syndecan-1 (clone B-A38, CellMarque) immunohistochemistry (IHC), respectively, according to standard procedures using an automated BenchMark ULTRA IHC/ISH staining platform (Ventana Medical Systems). IHC signals were detected with 3,3′-Diaminobenzidine (DAB) and counterstained with hematoxylin prior to coverslipping. H&E and IHC slides were digitalized at 20x using an Aperio CS2 Digital Pathology Scanner (Leica Biosystems). The immunohistofluorescence slides were imaged on a Vectra 2.0 multispectral slide imaging system (PerkinElmer) using 198 high-power fields with DAPI, FITC, and CY3 filters in place.

### Pathology reconfirmation and region of interest annotation

A board-certified surgical pathologist who was blinded to clinicopathological features and all quantitative data reviewed each core on the H&E stained section of the TMA to reconfirm the presence of cancer cells, PDAC histology, and suitable tissue quality. Using Aperio ImageScope viewing software (Leica Biosystems), normal ducts (from normal adjacent tissues) and PDAC ducts were annotated using a 400 × 400 μm region of interest (ROI) tool while ensuring that confounding tissue features (*i.e.* blood vessels, nerves, adipose tissue, smooth muscle cells of the duodenal wall, chronic pancreatitis) were omitted from the ROI. TMA cores that contained only fibrosis or necrotic debris and no cancer cells were omitted from analysis. To account for intra-tumoral PDAC heterogeneity the TMA was specifically designed to sample different areas of the tumors. Up to 4 cancer-containing cores (low grade cancer, high-grade cancer, tumor core and infiltrating edge) from each resection specimen were selected for incorporation into the TMA. To further control for intra-tumoral heterogeneity, we only analyzed patients that were represented by two or more confirmed PDAC cores so that data was not under or overestimated by tissue availability or tumor sampling bias. In addition, wherever possible, 2 different ROIs were identified per cancer-containing TMA core. Consequently, at least 4 and up to 8 different PDAC ROIs were examined for each patient.

### Characteristics of final confirmed patient cohort

The final patient cohort consisted of 114 patients represented by 83 normal adjacent, 84 low grade, 74 high grade, 99 tumor core, and 99 infiltrating tumor edge cores. 53 of the patients were female and 61 were male. The median age of diagnosis was 69.2 years (range 48 – 88 years). The median overall survival (OS was 24 months (range 12.2 – 125.6 months), and the overall 5-year survival rate was 14.9%. Other key clinicopathological features are shown in Supplementary Table S1.

### Second Harmonic Generation imaging, processing, and collagen alignment analyses

Individual TMA cores were imaged in entirety for SHG signal using a multiphoton system custom built by the Laboratory for Optical and Computational Instrumentation known as CAMM (Compact Automated Multiphoton Microscope). CAMM was designed with clinical translation in mind to rapidly screen large ROIs in histology samples for cancer-associated changes in collagen fiber properties [99]. A Ti:sapphire femtosecond laser was used to deliver 780 nm light to the tissue, and SHG signal was detected in the forward direction after spectrally filtering (390/18 BP, Semrock) using a 20x air objective (Nikon S Fluor NA = 0.75). All cores were acquired as a montage of 1024×1024 image tiles (10% overlap) using WiscScan software with consistent power and gain settings.

Individual SHG image tiles were stitched according to acquisition metadata to form a whole core composite image using FIJI [100]. Each H&E and IHC stained core was manually extracted from the Aperio files using Aperio ImageScope and converted to a Tagged Image Format File (.tiff) using Bio-Formats [101]. Whole core H&E brightfield and corresponding SHG images were registered using a two-step process. The first step was to extract collagenous stroma from H&E brightfield images by enhancing the colors using decorrelation stretch [102] and performing color separation using K-means clustering algorithm to find the best collagen estimate. The second step involved using an iterative intensity-based image registration algorithm to find the affine transform that registers the collagen extracted image to the SHG image. The algorithm was multi-resolution and started with the coarsest level of the images and uses the joint probability distribution of a sampling of pixels from two images to measure the certainty that the values of one set of pixels map to similar values in the other image. The algorithm then went to the next level of resolution to adjust the parameters from the previous step using a gradient descent method and continued this approach to the finest level of image resolution to find the final affine transform between the SHG image (template) and H&E image (source). This algorithm was modified to also register the serially-sectioned H&E and IHC cores on the basis of robust tissue features. All H&E pathology-marked ROIs were then transferred to each corresponding registered image and cropped to create a final image dataset.

Collagen fiber analysis was performed on SHG images using default settings in the CT-FIRE software package (http://loci.wisc.edu/software/ctfire) as previously described [43,59,103]. Alignment was calculated as the mean resultant vector length in circular statistics, which is based on the absolute fiber angles and spans a scale from 0.0 – 1.0 (1.0 indicates all fibers in the same direction).

### Immunohistostaining quantification

Percent positivity of α-SMA staining was defined as DAB pixels/(DAB + hematoxylin pixels) and determined using the Color Deconvolution FIJI plugin for hematoxylin and DAB [104]. Color vectors were manually constructed from single-stained control DAB and hematoxylin slides. Since syndecan-1 can be expressed by the stroma and epithelium, a semi-quantitative scale was used to exclusively assess the stromal compartment. Two observers, blinded to clinical outcome, interpreted the percentage of positive stromal cells as follows: 1, negative/low (<10%); 2, moderate (10-50%); 3, high (>50%). Staining intensity was not considered in the final score, and the two reviewer scores were averaged for each ROI. For fluorescently labeled specimens, a spectral library for tissue autofluorescence, DAPI, E-cadherin (488), and vimentin (555) was created in Nuance (version 3.0.2). InForm image analysis software (version 2.1) was subsequently used to create an algorithm that included the following steps: spectral unmixing and removal of autofluorescence, training and automatic segmentation of tumor epithelium on the basis of E-cadherin and DAPI, and scoring of individual tumor cells for the co-expression of E-cadherin and vimentin. All cores were individually reviewed for suitable stain, image, and epithelial segmentation quality.

### Statistical analyses

Statistical analyses were performed in GraphPad Prism (version 6) and R (version 3.2.3). Continuous data were analyzed with a Kruskal-Wallis (3 or more groups) or Mann-Whitney U (2 groups) based on Shapiro-Wilk normality test results. Spearman rank-order test was used to calculate the correlation between continuous variables. The association of collagen alignment with important clinicopathological characteristics was evaluated using Fisher's Exact test. For survival analyses, a patient value was computed by averaging all available ROI measurements from the tumor regions (low grade, high grade, core, and edge). Overall survival (OS) was defined as the time from diagnosis to death or the last follow-up examination (censored) for living patients. X-tile software (version 3.6.1) was used to determine the cutoff for dichotomizing high and low alignment patients [65]. A separate training cohort of 70 patient tissues from a commercial TMA (US Biomax, Inc., #HPan-Ade180Sur-01) was first analyzed to determine an optimal alignment cut-off value (0.60). This was subsequently validated to be significant in the final 114 patient experimental cohort. Kaplan-Meier survival curves were generated and the difference in survival curves was evaluated using the Log-rank test. Cox-regression multivariate proportional hazard analysis was performed to determine the independent prognostic significance of factors. A backward selection procedure with a *p*-value cutoff of <0.25 (determined by univariate analysis) was implemented to identify variables to include in the final multivariate model. We also included currently utilized clinical indicators (pT, pN, margin, grade) as covariates. Results are expressed as hazard ratios (HR) and 95% confidence intervals (CI). All *p*-values were calculated two-sided and >0.05 was considered significant.

## SUPPLEMENTARY FIGURES AND TABLE




