# Supplementary material for: Highly aligned stromal collagen is a negative prognostic factor following pancreatic ductal adenocarcinoma resection
Source: Oncotarget. 2016 Oct 20;7(46):76197–213. doi: 10.18632/oncotarget.12772 (PMC5342807; doi:10.18632/oncotarget.12772)
Supplement: Supplementary file 1 [file oncotarget-07-76197-s001.pdf]

Highly aligned stromal collagen is a negative prognostic factor following pancreatic ductal adenocarcinoma resection

SUPPLEMENTARY FIGURES AND TABLE

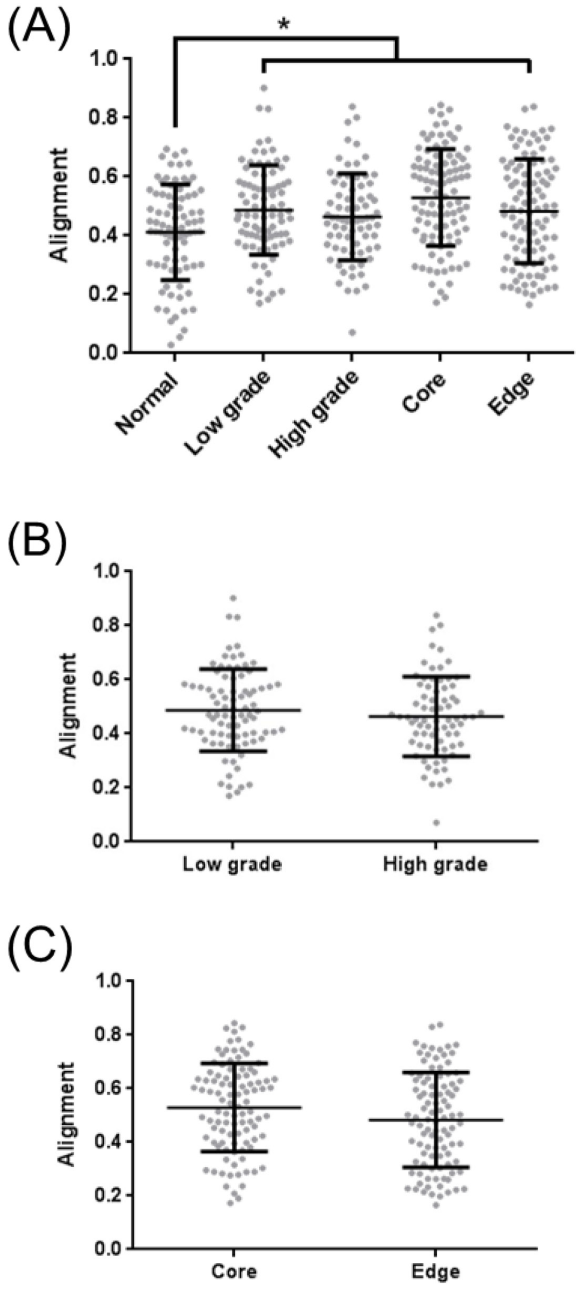

Supplementary Figure S1: Collagen alignment as a function of tissue type. A. Collagen alignment in the periductal stroma of different PDAC tissue regions versus normal. Each data point is representative of a patient core. N = 83 normal, 84 low grade, 74 high grade, 99 tumor core, 99 infiltrating edge cores. Bars indicate the mean ± SD. *$p < 0.05$. **B.** Collagen alignment in the periductal stroma of low versus high grade PDAC cells. Each data point is representative of a patient core. N = 84 low grade, 74 high grade cores. Bars indicate the mean ± SD. $p = 0.301$. **C.** Collagen alignment in the periductal stroma of the PDAC tumor core versus infiltrating edge. Each data point is representative of a patient core. N = 99 tumor core, 99 infiltrating edge cores. Bars indicate the mean ± SD. $p = 0.061$.

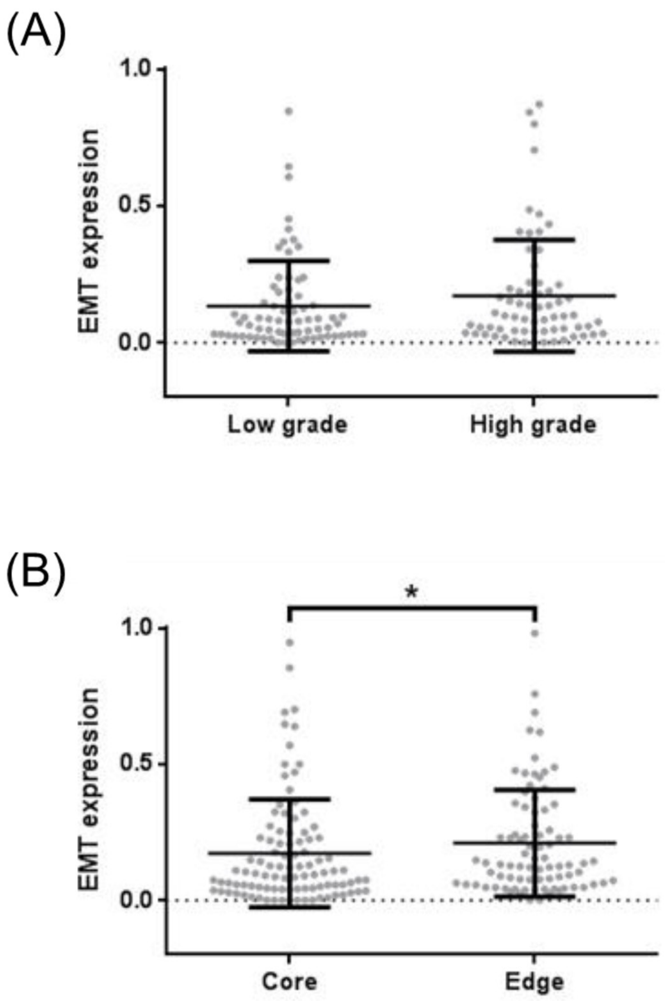

Supplementary Figure S2: EMT expression as a function of PDAC tumor region. A. EMT expression by low and high grade PDAC cells. Each data point is representative of a patient core. N = 70 low grade, 66 high grade cores. Bars indicate the mean ± SD. p = 0.188. **B.** EMT expression by PDAC cells in the tumor core and infiltrating edge. Each data point is representative of a patient core. N = 97 tumor core, 92 infiltrating edge cores. Bars indicate the mean ± SD. * p = 0.043.

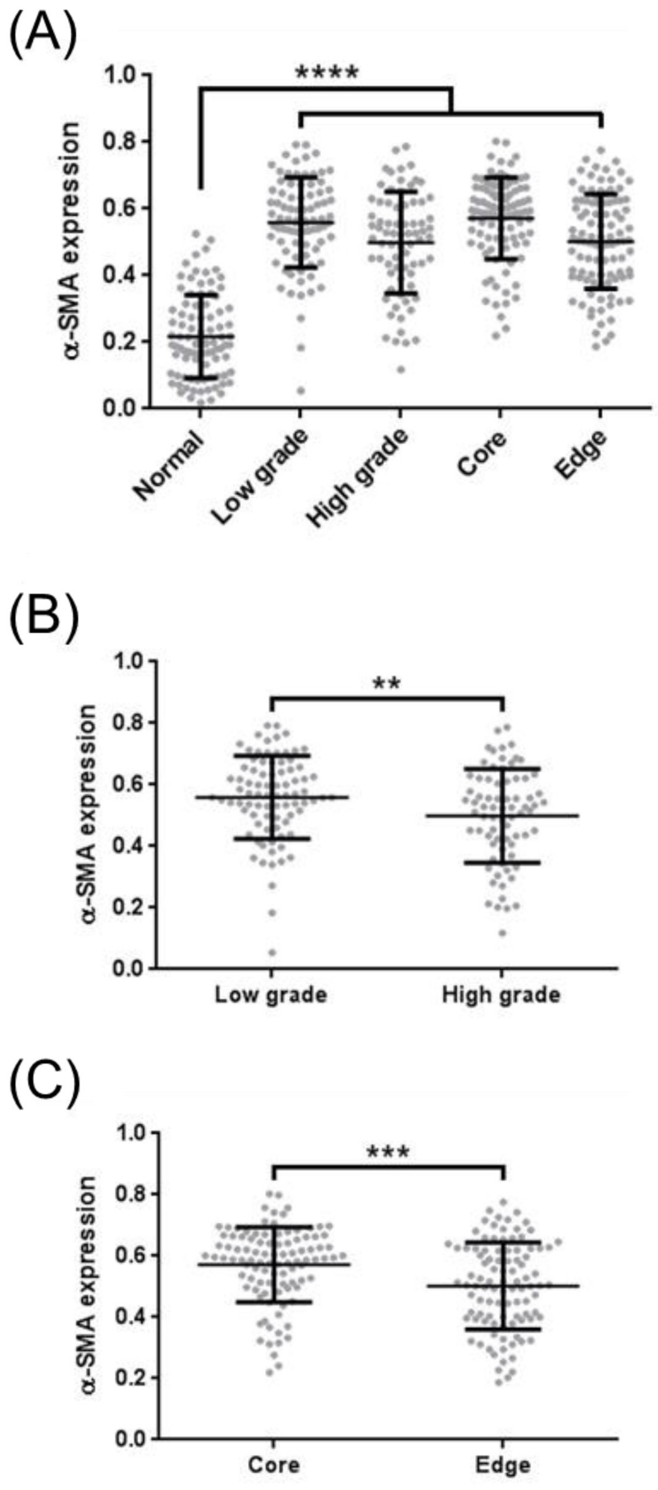

Supplementary Figure S3: α-SMA expression as a function of tissue type. A. α-SMA expression in the periductal stroma of different PDAC tissue regions versus normal. Each data point is representative of a patient core. N = 83 normal, 84 low grade, 74 high grade, 99 tumor core, 99 infiltrating edge cores. Bars indicate the mean ± SD. ****$p < 0.0001$. **B.** α-SMA expression in the periductal stroma of low and high grade PDAC cells. Each data point is representative of a patient core. N = 84 low grade, 74 high grade cores. Bars indicate the mean ± SD. **$p = 0.008$. **C.** α-SMA expression in the periductal stroma of PDAC cells in the tumor core and infiltrating edge. Each data point is representative of a patient core. N = 99 tumor core, 99 infiltrating edge cores. Bars indicate the mean ± SD. ***$p = 0.0004$.

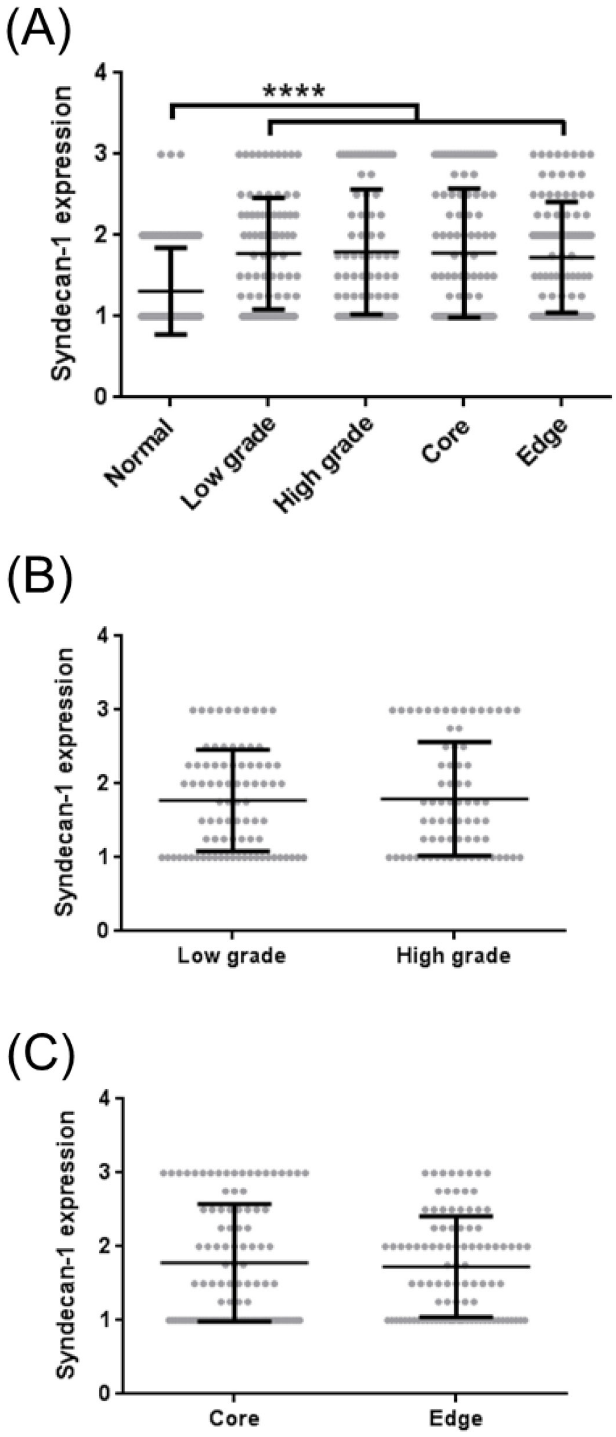

Supplementary Figure S4: Syndecan-1 expression as a function of tissue type. A. Syndecan-1 expression in the periductal stroma of different PDAC tissue regions versus normal. Each data point is representative of a patient core. N = 83 normal, 84 low grade, 74 high grade, 99 tumor core, 99 infiltrating edge cores. Bars indicate the mean ± SD. ****$p < 0.0001$. **B.** Syndecan-1 expression in the periductal stroma of low and high grade PDAC cells. Each data point is representative of a patient core. N = 84 low grade, 74 high grade cores. Bars indicate the mean ± SD. $p = 0.974$. **C.** Syndecan-1 expression in the periductal stroma of PDAC cells in the tumor core and infiltrating edge. Each data point is representative of a patient core. N = 99 tumor core, 99 infiltrating edge cores. Bars indicate the mean ± SD. $p = 0.805$.

Supplementary Table S1: Clinicopathological characteristics of final 114 patient study cohort.

See Supplementary File 1
