# Supplementary material for: Highly aligned stromal collagen is a negative prognostic factor following pancreatic ductal adenocarcinoma resection
Source: Oncotarget. 2016 Oct 20;7(46):76197–213. doi: 10.18632/oncotarget.12772 (PMC5342807; doi:10.18632/oncotarget.12772)
Supplement: Supplementary file 2 [file oncotarget-07-76197-s002.docx]

| **Characteristic** | **Category** | ***n*** |
| --- | --- | --- |
| Age (yr) | ≤65 | 40 |
|  | >65 | 74 |
| Gender | Female | 53 |
|  | Male | 61 |
| Race | Caucasian | 100 |
|  | Unknown | 4 |
| Tumor location | Head | 103 |
|  | Other | 11 |
| Tumor size (cm) | ≤2 | 24 |
|  | >2 | 89 |
|  | Unknown | 1 |
| pT | T1 | 9 |
|  | T2 | 24 |
|  | T3 | 76 |
|  | T4 | 4 |
|  | Unknown | 1 |
| pN | N0 | 33 |
|  | N1 | 81 |
| Stage | IA | 5 |
|  | IB | 7 |
|  | IIA | 20 |
|  | IIB | 77 |
|  | III | 4 |
|  | Unknown | 1 |
| Grade | G1 | 23 |
|  | G2 | 72 |
|  | G3 | 11 |
|  | Unknown | 8 |
| Venous invasion | No | 59 |
|  | Yes | 22 |
|  | Unknown | 33 |
| Lymphatic invasion | No | 52 |
|  | Yes | 29 |
|  | Unknown | 33 |
| Perineural invasion | No | 22 |
|  | Yes | 67 |
|  | Unknown | 25 |
| Surgery year | 1987-1998 | 14 |
|  | 1999-2005 | 35 |
|  | 2006-2012 | 65 |
| Margin | R0 | 86 |
|  | R1 | 28 |
| Adjuvant therapy | No | 45 |
|  | Yes | 69 |
